# Supplementary material for: Perceived safety and mental health among Asian American women: Exploring the moderating role of loneliness and resilience
Source: PLoS One. 2025 Nov 21;20(11):e0317322. doi: 10.1371/journal.pone.0317322 (PMC12637940; doi:10.1371/journal.pone.0317322)
Supplement: Attachment — Submitted filename: Review comments.docx [file pone.0317322.s001.docx]

Perceived Safety and Mental Health among Asian American Women: Exploring the Moderating Role of Loneliness and Resilience

**Introduction**

1. In lines 48-49, “*Existing literature suggests that this group, particularly Asian American women, face a disproportionate burden of mental health challenges.”*

If you're treating "group" as singular, you should use "faces" instead of “face”.

2. In lines 57-58, “*This largely stems from their marginalization based on intersectional identities as both Asian and women, rooted in a legacy of gendered racism.”*

To strengthen the statement’s credibility, appropriate citations and references should be included.

3. *In lines 106-108, “However, the influence of loneliness on the mental health of Asian American women, particularly in the context of environmental safety threats remains largely unexplored.”*

Your paragraph is generally clear. Reorganizing the sentences can improve logical flow. You may want to move this research gap sentence after the current research scope regarding the general Asian American community.

**Methods**

4. In lines 150-152, *“REDCap is a secure, HIPAA (Health Insurance Portability and Accountability Act) compliant software platform designed to support data collection for research studies.”*

It is important to cite the official description so that readers can understand its origin and validation

**Measures**

5. Even though you mentioned the reliability and validity of some measurements, it is still important to check and report the internal consistency (e.g., Cronbach’s alpha) of each measurement for your sample.

**Result**

6. In Line 245, “*Table 1. Sample Characteristics of Asian American Women (n=349)”*

Please follow APA 7th edition guidelines for table headings and formatting and avoid using Excel-style tables. (Apply these suggestions for other tables as well.)

One example is below:

“

**Table 1.**

*Sample Characteristics of Asian American Women (n=349) “*

7. In lines 269 to 271:
   The statement “Loneliness did not moderate the relationship between any avoidant behaviors and depression. Loneliness moderated the relationship between staying away from public spaces and anxiety” lacks clear statistical evidence to support these claims. I recommend including the corresponding p-values or related statistical measures in the text to substantiate these conclusions.
8. In lines 272 to 276:

Some adjusted odds ratios, such as aOR = 4.77, do not appear in Table 2 or Table 3. Please clarify which table or figure they are presented in and ensure that the numbering and explanations in the text align with the corresponding tables or figures. If these adjusted odds ratios are illustrated in Figure 1, please label them clearly to improve clarity for readers.

There is a mention of Figure 1 related to the interaction effects on anxiety, but it is not explained clearly. Please ensure that Figure 1 is clearly presented, with appropriate labeling of any significant numbers, and properly referenced in the text.

9. In lines 285:
   In the phrase “(all P>0.05),” the “P” should be italicized for consistency with statistical reporting conventions.

**Discussion/Conclusion**

10. In line 328 – Theoretical Background:
    You refer to the Person and Environment (P-E) fit theory in line 328, but it isn’t integrated into your research questions. Instead of addressing P-E fit theory in the discussion, I recommend adding a brief description of the P-E fit theory in the literature review or theoretical framework section to help guide the reader in understanding how this theory informs your study.
