# Supplementary material for: Perceived safety and mental health among Asian American women: Exploring the moderating role of loneliness and resilience
Source: PLoS One. 2025 Nov 21;20(11):e0317322. doi: 10.1371/journal.pone.0317322 (PMC12637940; doi:10.1371/journal.pone.0317322)
Supplement: Attachment — Submitted filename: 2025_09_17_Response to Reviewer_PLOS ONE_Clean.docx [file pone.0317322.s002.docx]

Dear Dr. Renata Komalasari,

We appreciate the opportunity to revise and resubmit our manuscript “*Perceived Safety and Mental Health among Asian American Women: Exploring the Moderating Role of Loneliness and Resilience*” to PLOS ONE. Please find our point-by-point responses below. The comments of editor and reviewers are indicated, and our responses immediately follow. Please also find a revised manuscript with tracked changes and a cleaned manuscript.

**Comments from editor and reviewers**

Journal Requirements:

When submitting your revision, we need you to address these additional requirements.

1. Please ensure that your manuscript meets PLOS ONE's style requirements, including those for file naming. The PLOS ONE style templates can be found at

<https://journals.plos.org/plosone/s/file?id=wjVg/PLOSOne_formatting_sample_main_body.pdf> and

[https://journals.plos.org/plosone/s/file?id=ba62/PLOSOne_formatting_sample_title_authors_affiliations.pdf](https://urldefense.com/v3/__https:/track.editorialmanager.com/CL0/https:*2F*2Fjournals.plos.org*2Fplosone*2Fs*2Ffile*3Fid=ba62*2FPLOSOne_formatting_sample_title_authors_affiliations.pdf/1/010f019860cf5ca3-ca75a06b-7eef-4da8-8349-2918323c26f8-000000/0vFNixpTgFw4ds-9WdlLaHR8MvdqiKzf-l9Gbhpdsrs=222__;JSUlJSUlJQ!!MXfaZl3l!bp9D8fCxKy3bvw0j1AxTX6PucR09ThG1tF8DXr82Bo3_stiSqHP5SlGrLxTvR66xMcS3mWNc2kjnWh9d9w8F-A$)

- **Response**: We have followed the PLOS ONE style requirement and ensured that our manuscript, including file naming and formatting, now fully conforms to the journal’s guidelines.

2. Thank you for stating in your Funding Statement:
“This work was supported by the National Institutes of Health (NIH) – National Institute on Minority Health and Health Disparities [grant number U54MD000538] and by the NIH – National Heart, Lung, and Blood Institute [grant number 1R01HL160324].”

Please provide an amended statement that declares *all* the funding or sources of support (whether external or internal to your organization) received during this study, as detailed online in our guide for authors at http://journals.plos.org/plosone/s/submit-now. Please also include the statement “There was no additional external funding received for this study.” in your updated Funding Statement.
Please include your amended Funding Statement within your cover letter. We will change the online submission form on your behalf.

- **Response**: Thank you and we have included the following amended Funding Statement to the cover letter: “This work was supported by the National Institutes of Health (NIH) – National Institute on Minority Health and Health Disparities [grant number U54MD000538],National Heart, Lung, and Blood Institute [grant number 1R01HL160324], and National Institute of Mental Health [grant number R34MH136914-01A1]. There was no additional external funding received for this study. The funders had no role in study design, data collection and analysis, decision to publish, or preparation of the manuscript.”

3. Thank you for stating the following financial disclosure:
“This work was supported by the National Institutes of Health (NIH) – National Institute on Minority Health and Health Disparities [grant number U54MD000538] and by the NIH – National Heart, Lung, and Blood Institute [grant number 1R01HL160324].”

Please state what role the funders took in the study. If the funders had no role, please state: "The funders had no role in study design, data collection and analysis, decision to publish, or preparation of the manuscript."
If this statement is not correct you must amend it as needed.
Please include this amended Role of Funder statement in your cover letter; we will change the online submission form on your behalf.

- **Response**: We have clarified that the funders were not involved in study design, data collection and analysis, decision to publish, or manuscript preparation in the cover letter. We have included the following amended Funding Statement to the cover letter: “This work was supported by the National Institutes of Health (NIH) – National Institute on Minority Health and Health Disparities [grant number U54MD000538],National Heart, Lung, and Blood Institute [grant number 1R01HL160324], and National Institute of Mental Health [grant number R34MH136914-01A1]. There was no additional external funding received for this study. The funders had no role in study design, data collection and analysis, decision to publish, or preparation of the manuscript.”

4. We note that you have indicated that there are restrictions to data sharing for this study. For studies involving human research participant data or other sensitive data, we encourage authors to share de-identified or anonymized data. However, when data cannot be publicly shared for ethical reasons, we allow authors to make their data sets available upon request. For information on unacceptable data access restrictions, please see <http://journals.plos.org/plosone/s/data-availability#loc-unacceptable-data-access-restrictions>.

Before we proceed with your manuscript, please address the following prompts:

a) If there are ethical or legal restrictions on sharing a de-identified data set, please explain them in detail (e.g., data contain potentially identifying or sensitive patient information, data are owned by a third-party organization, etc.) and who has imposed them (e.g., a Research Ethics Committee or Institutional Review Board, etc.). Please also provide contact information for a data access committee, ethics committee, or other institutional body to which data requests may be sent.

b) If there are no restrictions, please upload the minimal anonymized data set necessary to replicate your study findings to a stable, public repository and provide us with the relevant URLs, DOIs, or accession numbers. Please see <http://www.bmj.com/content/340/bmj.c181.long> for guidelines on how to de-identify and prepare clinical data for publication. For a list of recommended repositories, please see <https://journals.plos.org/plosone/s/recommended-repositories>. You also have the option of uploading the data as Supporting Information files, but we would recommend depositing data directly to a data repository if possible.

Please update your Data Availability statement in the submission form accordingly.

- **Response**: We have updated the Data Availability statement to include an institutional contact for data access. NYULH IRB Operations: IRB-Info@NYULangone.org

5. If the reviewer comments include a recommendation to cite specific previously published works, please review and evaluate these publications to determine whether they are relevant and should be cited. There is no requirement to cite these works unless the editor has indicated otherwise.

- **Response**: Thank you!

6. Please review your reference list to ensure that it is complete and correct. If you have cited papers that have been retracted, please include the rationale for doing so in the manuscript text, or remove these references and replace them with relevant current references. Any changes to the reference list should be mentioned in the rebuttal letter that accompanies your revised manuscript. If you need to cite a retracted article, indicate the article’s retracted status in the References list and also include a citation and full reference for the retraction notice.

- **Response**: We have carefully reviewed our reference list to ensure accuracy. In response to reviewer concerns, we have added several new references to strengthen the clarity and rigor of the manuscript; these additions are detailed and justified in our responses to specific reviewer comments. None of the references cited in our manuscript have been retracted.

**Additional Editor Comments:**

Thank you for the opportunity to handle this manuscript. I have read the text with interest and have some questions for you and issues that I would like you to address:

1. Please provide coding for categorical variables to help with interpretation of logistic regression results.

- **Response**: Thank you and we have now specified how categorical variables were coded in the measures section. See updated line 186-187; 194-195; 202-203; 213-214; 220; 228-229.

2. Lines 271 - 273: The authors cited an aOR = 4.77, 95% CI: 1.24, 18.34, showing that among women who did not feel lonely, those who stayed away from public spaces due to safety concerns had 4.77 times the odds of reporting anxiety compared to those who did not stay away. Are you referring to aOR value of 4.81 as reported in in Table 3? Is yes, which value is correct: 4.77 or 4.81?

- **Response:** Thank you for pointing this out. To clarify, the value of 4.81 reported in Table 3 is the Wald chi-square statistic for the interaction term (*stay away from public spaces × loneliness*) with the corresponding *p* value of 0.028, which was presented to indicate statistical significance of the interaction. The aOR = 4.77, 95% CI: 1.24–18.34 represents the conditional effect of staying away from public spaces on anxiety among women who did not report loneliness. These conditional effect estimates are not displayed in Table 3 but are reported in-text to illustrate how the moderation effect of loneliness operates. We have revised the Results section to explicitly note this distinction for clarity.

3. Lines 273-275 Is this statement refers to the simple slope analysis interpretation. If yes, I suggest indicating this in the sentence. The authors stated that among women who were lonely, staying away from public spaces was associated with lower odds of reported anxiety compared to those who did not stay away. The value of aOR = 0.93 was cited but I did not see such value in Table 3 as the authors indicated. However, learning from simple slope graph in Fig 1, my thought is that among those who were lonely there was no differences in anxiety reports between those who stayed away from public spaces or not. Hence, no moderating effect was observed amongst women who were lonely. Therefore, the sentence "Loneliness moderated the association between avoiding public spaces and anxiety" found in the abstract (line 37) and in line 271 may be misleading as moderation effect of loneliness was observed only among women who were not lonely. Please ensure correct interpretation of statistical results across the paper.

- **Response:** Thank you for this thoughtful comment. We have clarified in the revised manuscript that we are reporting results from a simple slopes (conditional effects) analysis. Specifically, the estimates (aOR = 4.77, 95% CI: 1.24–18.34; aOR = 0.93, 95% CI: 0.53–1.64) are conditional effect estimates that were not presented in Table 3, which instead reports the Wald chi-square test for the interaction terms.
- We agree that the figure suggests no significant differences among women who reported loneliness, consistent with the conditional estimate (aOR = 0.93, 95% CI: 0.53–1.64, not statistically significant). By definition, moderation is present when the association between an independent variable and an outcome differs across levels of a moderator. In our study, the effect of staying away from public spaces on anxiety differed by loneliness status: it was significant among women who were not lonely but not significant among those who were lonely. We have now revised the Results and abstract to clearly explain the nature of the moderation effect of loneliness.

4. Please follow APA format in reporting statistical reports: for example: please use small letter 'p' in reporting p value, instead of capital P.

- **Response:** Thank you and we have updated the statistical reporting to align with APA 7th formatting, including non-capitalized and italicized p value.

**Reviewers' comments:**

**Review Comments to the Author**

Please use the space provided to explain your answers to the questions above. You may also include additional comments for the author, including concerns about dual publication, research ethics, or publication ethics. (Please upload your review as an attachment if it exceeds 20,000 characters)

**Reviewer #1: Introduction**
1. In lines 48-49, “Existing literature suggests that this group, particularly Asian American women, face a disproportionate burden of mental health challenges.”
If you're treating "group" as singular, you should use "faces" instead of “face”.

- **Response**: Thanks so much for noting this. We have changed “face” to “faces”.

2. In lines 57-58, “This largely stems from their marginalization based on intersectional identities as both Asian and women, rooted in a legacy of gendered racism.”
To strengthen the statement’s credibility, appropriate citations and references should be included.

- **Response**: Thank you and we have added citation to support our statement.
- Forbes N, Yang LC, Lim S. Intersectional discrimination and its impact on Asian American women’s mental health: A mixed-methods scoping review. Front Public Health [Internet]. 2023. Available from: https://www.frontiersin.org/articles/10.3389/fpubh.2023.993396

3. In lines 106-108, “However, the influence of loneliness on the mental health of Asian American women, particularly in the context of environmental safety threats remains largely unexplored.”
Your paragraph is generally clear. Reorganizing the sentences can improve logical flow. You may want to move this research gap sentence after the current research scope regarding the general Asian American community.

- **Response**: Thank you, we have now moved this sentence to follow the discussion of existing evidence on the general Asian American community to enhance the logical flow.

Methods
4. In lines 150-152, “REDCap is a secure, HIPAA (Health Insurance Portability and Accountability Act) compliant software platform designed to support data collection for research studies.”
It is important to cite the official description so that readers can understand its origin and validation

- **Response**: Thank you for this suggestion. We have added the official REDCap references to acknowledge the origin of the platform: “REDCap is a secure, HIPAA (Health Insurance Portability and Accountability Act) compliant software platform designed to support data collection for research studies (47,48)”

47. Harris PA, Taylor R, Minor BL, Elliott V, Fernandez M, O’Neal L, et al. The REDCap consortium: Building an international community of software platform partners. J Biomed Inform. 2019;95:103208.

48. Harris PA, Taylor R, Thielke R, Payne J, Gonzalez N, Conde JG. Research electronic data capture (REDCap)—A metadata-driven methodology and workflow process for providing translational research informatics support. J Biomed Inform. 2009;42(2):377–81.

Measures
5. Even though you mentioned the reliability and validity of some measurements, it is still important to check and report the internal consistency (e.g., Cronbach’s alpha) of each measurement for your sample.

- **Response**: Thank you for this helpful suggestion. We have now reported the internal consistency (Cronbach’s α) for each measurement scale within our sample in the Measures section, which also complements the psychometric information on the scales. The UCLA Loneliness Scale (3-item), The Brief Resilience Scale, PHQ-9, and GAD-7 all demonstrated good to excellent internal consistency (αs = .82–.91).

Result
6. In Line 245, “Table 1. Sample Characteristics of Asian American Women (n=349)”

Please follow APA 7th edition guidelines for table headings and formatting and avoid using Excel-style tables. (Apply these suggestions for other tables as well.)
One example is below:
“Table 1. Sample Characteristics of Asian American Women (n=349) “

- **Response**: Thank you for noting this. We reviewed the PLOS ONE formatting guidelines, which specifically require that tables be created as cell-based tables in Microsoft Word or embedded from Microsoft Excel. Because of these journal requirements, we did not modify the table formatting: <https://journals.plos.org/plosone/s/file?id=wjVg/PLOSOne_formatting_sample_main_body.pdf>. However, within these journal formatting constraints, we ensured that table titles and notes adhere to APA 7th edition style conventions whenever possible.

7. In lines 269 to 271:
The statement “Loneliness did not moderate the relationship between any avoidant behaviors and depression. Loneliness moderated the relationship between staying away from public spaces and anxiety” lacks clear statistical evidence to support these claims. I recommend including the corresponding p-values or related statistical measures in the text to substantiate these conclusions.

- **Response**: Thank you for noting this. We have clarified the statistical evidence supporting our conclusions: “Loneliness did not moderate the relationship between any safety-related behavioral modifications and depression (all *p*> .05). However, it moderated the relationship between staying away from public spaces and anxiety (See *Table 3 and* *Fig 1*). The Wald chi-square statistic for this interaction term was 4.81 (*p*= .028), indicating a significant moderation effect.”

8. In lines 272 to 276:
Some adjusted odds ratios, such as aOR = 4.77, do not appear in Table 2 or Table 3. Please clarify which table or figure they are presented in and ensure that the numbering and explanations in the text align with the corresponding tables or figures. If these adjusted odds ratios are illustrated in Figure 1, please label them clearly to improve clarity for readers.
There is a mention of Figure 1 related to the interaction effects on anxiety, but it is not explained clearly. Please ensure that Figure 1 is clearly presented, with appropriate labeling of any significant numbers, and properly referenced in the text.

- **Response**: Thank you. We have clarified in the Results section that the adjusted odds ratios (aORs) of 4.77 and 0.93 are conditional effect estimates of the interaction between loneliness and staying away from public spaces on anxiety. These values are not presented in Table 3 because Table 3 displays only the Wald chi-square statistic and *p* value for the interaction term to indicate its statistical significance. To avoid confusion, we now explicitly state in the text that these conditional odds ratios are reported in the manuscript narrative but not in the tables. We have also revised the text to improve alignment with Figure 1 by explaining that the figure illustrates the moderation effect, showing the differential associations of staying away from public spaces with anxiety for lonely and non-lonely women. We have also updated the figure caption to note that the conditional odds ratios are reported in-text (not displayed on the figure itself) and clearly labeled the interaction effect in Figure 1.

9. In lines 285:
In the phrase “(all P>0.05),” the “P” should be italicized for consistency with statistical reporting conventions.

- **Response**: Thank you. We have thoroughly revised the manuscript to ensure that p values are italicized (in line with APA 7th edition guidelines) and not capitalized.

Discussion/Conclusion
10. In line 328 – Theoretical Background:
You refer to the Person and Environment (P-E) fit theory in line 328, but it isn’t integrated into your research questions. Instead of addressing P-E fit theory in the discussion, I recommend adding a brief description of the P-E fit theory in the literature review or theoretical framework section to help guide the reader in understanding how this theory informs your study.

- **Response**: Thank you for this thoughtful suggestion. The two theoretical perspectives referenced in the Discussion—including Person–Environment (P–E) fit theory—are intended as interpretive lenses to help situate our findings within broader conceptual frameworks. They were not guiding frameworks for the study design or research questions, which is why they were not introduced earlier in the manuscript. To avoid confusion, we have revised the text in the Discussion to clarify their role as post hoc interpretive tools rather than foundational theories. We also considered moving the P–E fit theory into the introduction but decided against it, as it was not used to guide hypotheses or analysis. Instead, we now provide clearer language to explain why these perspectives are included in the Discussion and how they help contextualize the study findings: “We draw on the Person and Environment (P-E) fit theory as a conceptual lens to understand these relationships, which posits that stress and adverse mental health outcomes arise when the environment fails to meet individual needs (69).”

**Reviewer #2: SUMMARY:**

In the wake of a rise in anti-Asian violence in the United States, the present study explores potential impact of perceived safety (across three types of locations) and behavioral changes due to safety concerns (as independent variables) upon depression and anxiety (as dependent variables) for Asian American women, with self-reported resilience and loneliness explored as potential moderating variables. From a convenience sample of n-345 Asian American Women, findings support a significant positive correlations between most of the independent variables and depression, anxiety, or both. The moderating variable of loneliness is only found to have significant influence on anxiety for respondents who stay away from public spaces (a behavioral change due to safety concern). Resilience is not found to have any significant influence on depression or anxiety. Authors elaborate on their methods, theoretical contexts, and potential interventions from these findings.

1. The manuscript is technically adequate for reporting the study as designed, yet demonstrates methodological weaknesses that go as far back as the design and analysis stages. I recommend the authors start over with more rigorous and informed analysis.

My greatest concern is that even though disaggregated demographic data has been collected, it is mentioned late and infrequently, then omitted from analysis without explanation or justification. Asian American women are not a monolith (I am thinking here of decolonial work by Rosalind S. Chou, as well as Aihwa Ong's "Cultural Citizenship as Subject-Making"); while a case could be made that perceptions of safety are more linked to how one is stereotyped in the world than one's own experience, this paper does not make any such case. There is no indication that demographics (including but not limited to ancestral nationality) were given much consideration at all during analysis; readers are given inadequate demographic descriptives and then told they were omitted from analysis due to lack of significance. The authors also seem to be unaware of the terms or value of convenience sampling or weighted data (cf. lines 435-441), so their snowball sample is not compared to any broader population figures at all. As a convenience sample, my previous point is all the more glaring: generalizability was never going to be strong, so the quality of the data lies in relationships that are largely unexplored here. No effort is made to justify the omission, let alone explore possible interactions with other findings. Without a more thorough report and contextualization of sample demographics -- ancestry and time in the U.S. most urgently -- readers can hardly surmise whether further items (such as regional and urban/suburban/rural distribution) might be missing.

- **Response**: We appreciate the reviewer’s emphasis on the importance of disaggregated demographic reporting and contextualization. We have carefully considered this feedback and substantially expanded both the scope and transparency of our demographic reporting.

**Expanded demographic reporting**

In the Measures section, we now include the full set of response categories for each sociodemographic variable exactly as presented to participants (e.g., all ethnic subgroup options, language categories, sexual orientation categories, education levels, and employment statuses). This addition improves clarity and transparency for readers by allowing them to see exactly how each variable was operationalized. In the Results section, we provide a detailed demographic table that includes ancestral subgroup (full breakdown in revised Table 1 and table notes). For all continuous variables, including age and years in the U.S., we now report the mean, standard deviation, and range.

**Inclusion of demographics in analysis**

We have clarified in the Methods section that these sociodemographic variables were pre-selected as control variables based on prior literature linking them to depression and anxiety among Asian American populations (Refs 61–63). We conducted exploratory analyses testing interactions between key demographics (ancestry, years in the U.S.) and the main independent variables in their relationships to depression and anxiety. Specifically, we examined interaction terms such as Perceived safety × ancestry, Perceived safety × years in the U.S., Behavioral changes × ancestry and Behavioral changes × years in the U.S. With few exceptions, these interactions were non-significant, indicating consistent associations across groups (See tables below). These exploratory findings are not the focus of the current study but are acknowledged in the limitations section.

| **Interaction terms** | ***Depression*** | | ***Anxiety*** | |
| --- | --- | --- | --- | --- |
|  | ***Wald Chi-square statistics*** | ***P-value*** | ***Wald Chi-square statistics*** | ***P-value*** |
| Feel unsafe in public space *** *Asian American groups* | 1.61 | 0.205 | 0.06 | 0.800 |
| Feel unsafe in public transportation*** *Asian American groups* | 1.38 | 0.241 | 1.15 | 0.284 |
| Feel unsafe in neighborhood *** *Asian American groups* | 4.11 | 0.043 | 2.44 | 0.118 |
| Stay away from public spaces *** *Asian American groups* | 0.004 | 0.948 | 0.66 | 0.418 |
| Avoid leaving home *** *Asian American groups* | 0.44 | 0.505 | 1.20 | 0.274 |
| Avoid transportation *** *Asian American groups* | 0.92 | 0.337 | 1.92 | 0.166 |
| Accompany *** *Asian American groups* | 0.45 | 0.500 | 0.02 | 0.878 |

| **Interaction terms** | ***Depression*** | | ***Anxiety*** | |
| --- | --- | --- | --- | --- |
|  | ***Wald Chi-square statistics*** | ***P-value*** | ***Wald Chi-square statistics*** | ***P-value*** |
| Feel unsafe in public space *** *Years in the US* | 3.96 | 0.047 | 0.02 | 0.898 |
| Feel unsafe in public transportation*** *Years in the US* | 0.09 | 0.768 | 1.52 | 0.217 |
| Feel unsafe in neighborhood *** *Years in the US* | 2.47 | 0.116 | 0.16 | 0.690 |
| Stay away from public spaces *** *Years in the US* | 0.04 | 0.847 | 0.66 | 0.415 |
| Avoid leaving home *** *Years in the US* | 0.05 | 0.825 | 0.55 | 0.459 |
| Avoid transportation *** *Years in the US* | 0.04 | 0.841 | 0.30 | 0.584 |
| Accompany *** *Years in the US* | 0.07 | 0.794 | 1.02 | 0.313 |

We also clarify that demographic variables were not omitted from the analyses. In our multivariable models, we controlled for demographic factors that were statistically significant at the level of 0.05 in bivariate analyses for the respective outcomes. Specifically: For all models with the outcome depression, we controlled for age, sexual orientation, education, employment, and language spoken at home. For all models with the outcome anxiety, we controlled for age, sexual orientation, employment, and language spoken at home. This approach ensured that relevant covariates were retained for each model, while avoiding overfitting given the sample size and number of predictors.

**Sampling**

We also clarify that our study did not employ snowball sampling; recruitment relied on convenience sampling through community-based outreach and online recruitment. We are aware of the implications of convenience sampling for generalizability and of the potential role of population weighting. As is typical with non-probability samples, weighting was not applied; however, we have explicitly acknowledged these limitations in the *revised Limitations* section and emphasized that our findings are not intended to be representative of all Asian American women. We highlight in the *revised* Limitations section that our sample, largely women with higher educational attainment and English proficiency, offers unique insights, while Asian American women with lower education or limited English proficiency who have more restrictive access to resources may face even greater mental health challenges. This study directly counters the “model minority” stereotype by showing that even highly educated and English-proficient Asian American women report substantial mental health burdens.

2. Perhaps most troubling is the authors' murky claims of causality. Measures are introduced and somewhat described, but no grounds are given for the causal relationship as examined -- in fact, the Discussion once implies an inverted causality (lines 356-359) and elsewhere implies no causality exists at all (line 428). No statistics or sources are given for potential overlap between depression and anxiety, nor between resilience and loneliness.

- **Response**: We appreciate the opportunity to clarify our rationale and the importance of addressing directionality. Given the cross-sectional nature of our study, we cannot establish causal relationships. Instead, our analyses focus on associations between perceived safety, behavioral adaptations, and mental health outcomes. In the Discussion, we intentionally acknowledged the possibility of bidirectional relationships (e.g., safety-related behavioral modifications may contribute to depressive/anxiety symptoms, while individuals with mood or anxiety disorders may also be more likely to adopt these behaviors). We view this approach as critical for transparency and for framing our findings within the study’s methodological limitations.

Regarding the reviewer’s concern about overlap between constructs: in our study, depression and anxiety were conceptualized and modeled as distinct outcomes of interest, consistent with prior research that treats these as separate clinical constructs with unique implications. Although comorbidity is well-documented, our aim was to examine independent associations of perceived safety and behavioral responses with each outcome. Similarly, loneliness and resilience were treated as distinct moderators to capture their respective roles. We agree that overlap among these constructs is important; but a full exploration is beyond the scope of the present study and not aligned with our research aims.

3. One more fundamental flaw: authors imply (lines 193-195) that the survey was cut short for respondents who reported feeling safe, and this lost data undermines the analysis; these responses could provide a vital control for internal and external comparison, and without them the data is incomplete for whatever portion (unreported) describe no unsafe feelings. (That descriptive statistics are not reported fully enough for me to tell if my reading is accurate is also an issue.)

- **Response**: We appreciate the opportunity to clarify this point. Our study specifically aimed to examine behavioral modifications made in response to perceived safety concerns. Accordingly, questions regarding behavioral modifications were administered only to participants who reported feeling less than "very safe" in any of the following contexts: public spaces, transportation, neighborhood. This skip logic was a deliberate design choice to ensure that reported behavioral modifications were meaningfully tied to safety-related perceptions, rather than general behavioral tendencies.

Importantly, all participants (N = 349) completed the full survey except for the behavior modification items, which were asked conditionally on reported safety concerns. Participants who reported feeling “very safe” were not presented with behavior modification questions, as it would not be conceptually appropriate to ask individuals without safety concerns whether they modified their behaviors due to safety concerns. In our sample, this represented only 7 participants. The remaining 342 participants were asked about safety-related behavioral modifications, providing a robust analytic sample.

We now clarified this approach with further details in the Methods section. Additionally, we have added the number of respondents who perceived their environment as unsafe and were therefore asked about behavior changes, to provide greater transparency in the Results section.

- Now in the measure section:
  - “*Behavior changes due to safety concerns. Only participants who did not perceive public spaces, public transportation, and neighborhoods as very safe were further asked whether they had ever changed their behaviors due to safety concerns (Yes/No). This includes four specific behaviors: staying away from public spaces, avoiding leaving their home, decreasing or avoiding the use of public transportation, and having someone accompany them when going outside (55). This decision was based on our study aim to examine behavioral modifications specifically in response to perceived safety concerns*.”

•	Now in the results section:

- “*Among Asian American women who perceived public spaces, transportation, or their neighborhood as unsafe (n=342), nearly half modified their behaviors due to safety concerns (not mutually exclusive): 54.4% avoided public spaces, 42.4% refrained from leaving home, 65.7% avoided using transportation, and 46.5% ensured they had someone with them when outside*.”

4. I have secondary concerns as well.

The authors adequately contextualize their research question amid a rise of anti-Asian violence, but do not mention whether things may have changed as the public conscience has moved away from COVID-19. If such data does not exist yet, so be it, but it warrants mention, especially in Discussion. Neither is there adequate elaboration about why the sample includes all Asian Americans instead of only those of East Asian ancestry, even though the distinction is raised on lines 239-240 and, to the best of my knowledge, highly relevant to COVID-era hate crimes.

- **Response**: Thanks for this important point. We agree that it is critical to acknowledge the ongoing nature of anti-Asian violence beyond the peak of the COVID-19 pandemic. To address this, we have added recent data to the Introduction showing that anti-Asian incidents surged by 59% in December 2024 and show no signs of declining. This contextualizes the continued relevance of our research topic and supports the need to examine the enduring mental health consequences among Asian American women.
- In response to the comment about the inclusion of all Asian subgroups in our sample, we have revised the Introduction to highlight that national data show similarly high rates of racism, discrimination, and violence across East, Southeast, and South Asian women. Specifically, 72% of East Asian, 75% of Southeast Asian, and 73% of South Asian women reported experiencing racism and/or discrimination; 35%, 37%, and 40% reported sexual harassment; and 9% of East and Southeast Asian women and 18% of South Asian women reported gender- or race-based physical violence. This evidence supports our decision to include women from diverse Asian subgroups, as the impacts of Anti-Asian are not limited to East Asian women alone. Unfortunately, regardless of how Asian women identify in terms of ethnic subgroup, they are perceived as the same; COVID-19 related discrimination, although, should theoretically be targeted only towards Chinese women, was directed to all Asian women regardless of ethnic subgroup.

5. There is a recurring lack of clarity as to when authors have chosen a tool, cutoff, or theoretical tangent because it has been recommended in other research versus their own whims and convenience. There is not necessarily a wrong answer, but authors must give one; examples include why the cutoff for depression measurement is different from the cutoff for anxiety measurement, the use of an unspecified "pre-selected list" (mentioned in lines 222-224 but notably not during Methods), and two theoretical frameworks introduced in discussion with neither buildup nor application. Many times, a comparison is made (examples include "high prevalence", line 370, or "disproportionate", line 334, but there are many others) without specifying who is being compared: general population, American women in general, Asian Americans in general, existing research of similar samples, or other groupings within this study? Authors must be explicit every time.

- **Response:** We appreciate the opportunity to clarify and respectfully note that the Measures section provides detailed justification for each measurement tool and associated cutoff point, based on established literature and/or validation studies. Related sources are cited throughout to support these decisions. For instance, both the PHQ-9 and GAD-7 are scored and interpreted using validated thresholds (≥10) to indicate moderate or greater symptom severity, which have been widely used in mental health research and validated in Asian American populations (Refs 46–51). Similarly, the cutoffs used for loneliness and resilience are based on prior peer-reviewed studies or original scale developers. We have reviewed the section again and added clarifying language wherever helpful, but believe our choices were methodologically sound and consistent with best practices.
- We have clarified in the Methods section that the sociodemographic variables, including age, nativity, years in the U.S., Asian ethnic group, sexual orientation, language spoken at home, education, and employment, were pre-selected based on prior literature demonstrating their relevance to depression and anxiety among Asian American populations (refs 61–63). This revision ensures greater transparency and grounds our variable selection in empirical evidence.
- The two theoretical perspectives referenced in the Discussion are intended as interpretive lenses to help readers situate our findings in broader conceptual/ theoretical context. They were not guiding frameworks for study design or analysis, which is why they were not introduced earlier in the introduction. We have revised the text to avoid confusion.
- We carefully reviewed the manuscript to ensure that all group comparisons were clearly specified where appropriate. In instances where no comparison group was available, we revised the language (e.g., replacing “disproportionate” with more terms such as “substantial”) to improve clarity and consistency for readers.

6. I think a lot more descriptives should be included, as detailed above, and there are technical flaws in how several statistics are reported, but the statistics themselves seem fine as presented. The statistics were not made available to reviewers for examination.

- **Response:** Thank you. All key descriptive statistics are now included in the manuscript tables and text so that readers and reviewers have full access to the results underlying our interpretations. Specifically, (1) we added detailed demographic information, perceived safety, safety-related behavioral modifications, moderators to the revised Table 1, including full distributions for all categorical variables and descriptive statistics (mean, standard deviation, and range) for continuous variables. (3) we carefully reviewed and corrected statistical reporting across the manuscript to ensure that they are following the APA 7^th^ format.

7. The manuscript is rough and would warrant a complete outside copyedit; the most egregious errors are inconsistency of own terms (e.g. "perceived safety" vs. "safety concerns", "behavioral modifications" vs. "modified behaviors") and capitalization that defies statistical convention (for example, "p-value" should never be capitalized because authors are discussing a sample -- capital letters reflect population-level data), but two paragraphs (lines 204-212 and lines 290-302) are sufficiently convoluted and unprofessional as to undermine the study itself. The authors seem to lack both technical knowledge (such as how to insert special characters like "≥" in Table 1, Education) and sensitivity to not "other" respondents whose responses were not listed (Table 1, Sexual Orientation). My preference that the analysis address mixed race and transgender identities is, admittedly, unlikely to provide statistically significant findings, but the present items still fail to meet minimal standards because almost none of the demographic items collected specify how they were structured in the survey: were respondents given a list, a blank space, or a combination of the two? Several times, figures cited from other studies fail to specify whether they report a similar population (Asian American women) or an adjacent one (Asian Americans at large, women in the U.S., etc.).

- **Response**: Thank you. We have revised the manuscript to ensure consistent terminology. Specifically, we clarified that perceived safety in our study refers to feeling safe in public spaces, transportation, and neighborhoods. In addition, we consistently use the term safety-related behavioral modifications throughout the manuscript to describe behavioral changes made in response to safety concerns. We also corrected all statistical reporting to be consistent with APA 7th edition (e.g., “*p*-value” in lowercase, the use of “≥”). We clarified how each demographic variable was measured in the methods section to provide transparency: “Sociodemographic information including age, nativity (US-born: yes/no), years in the US, Asian ethnic groups (What is your ancestry or ethnic identity? Please check all that apply [multiple responses allowed]: Bangladeshi, Burmese, Bhutanese, Cambodian, Chinese, Filipino, Hmong, Indian, Indonesian, Japanese, Korean, Laotian, Malaysian, Mongolian, Nepali, Okinawan, Pakistani, Sri Lankan, Taiwanese, Thai, Vietnamese, or Other [please specify]), sexual orientation (straight/lesbian or gay/bisexual/other), and language spoken at home (English/Asian language/English and another Asian language equally/Other [please specify]), education (never attended school or only attended kindergarten/grades 1–8/grades 9–11/grade 12 or general educational development/college 1–3 years/college 4 years or more) and employment (yes, part-time [<35 hours per week]/yes, full-time [>35 hours per week]/no, not employed) were also collected. These variables were pre-selected based on established literature on their relevance to mental health outcomes of interests (i.e., depression and anxiety) among Asian American populations (60–62).”
- We have also revised the two noted sections (lines 204–212 and lines 290–302) to improve clarity and precision of the sentences. Finally, when citing external statistics, we now specify the study population (e.g., elder Asian Americans) to avoid ambiguity.

8. The Discussion is deeply flawed, introducing almost as many new concepts as the introduction and making assertions that overstate or outright contradict the findings as reported (lines 394-395 are probably the worst). Several of the finest passages in the text (lines 321-323, 337-341, 366-369, and 382-387) are misplaced here and buried between passages of unscientific commentary.

- **Response**: We thank the reviewer for this critique. We have substantially revised the Discussion to enhance clarity, flow, coherence, and alignment with our reported results. In the revision, we retained and emphasized the strongest sections that directly interpret our results, while rephrasing or removing passages that could be misinterpreted as speculative or causal. We also revised lines 394–395 and other relevant sentences to avoid any language suggesting causal inference or contradictions with the results. In addition, we streamlined the Discussion to prevent the introduction of new concepts post hoc and to focus instead on interpreting our findings within the scope of the study and its limitations.

I applaud the authors for their hard work, their successful research grant, and their data collection. I hope this feedback leads to strong works in the future.

- **Response:** Thank you!
